# Omnichannel Customer Behavior: Key Drivers of Technology Acceptance and Use and Their Effects on Purchase Intention

**DOI:** 10.3389/fpsyg.2016.01117

**Published:** 2016-07-28

**Authors:** Emma Juaneda-Ayensa, Ana Mosquera, Yolanda Sierra Murillo

**Affiliations:** Departamento de Economía y Empresa, Universidad de La RiojaLogroño, Spain

**Keywords:** omnichannel experience, shopping motives, consumer behavior, omnishopper, fashion retail, technology acceptance model, UTAUT2

## Abstract

The advance of the Internet and new technologies over the last decade has transformed the retailing panorama. More and more channels are emerging, causing consumers to change their habits and shopping behavior. An omnichannel strategy is a form of retailing that, by enabling real interaction, allows customers to shop across channels anywhere and at any time, thereby providing them with a unique, complete, and seamless shopping experience that breaks down the barriers between channels. This paper aims to identify the factors that influence omnichannel consumers' behavior through their acceptance of and intention to use new technologies during the shopping process. To this end, an original model was developed to explain omnichannel shopping behavior based on the variables used in the UTAUT2 model and two additional factors: *personal innovativeness* and *perceived security*. The model was tested with a sample of 628 Spanish customers of the store Zara who had used at least two channels during their most recent shopping journey. The results indicate that the key determinants of purchase intention in an omnichannel context are, in order of importance: *personal innovativeness, effort expectancy*, and *performance expectancy*. The theoretical and managerial implications are discussed.

## Introduction

In recent years, advances in technology have enabled further digitalization in retailing, while also posing certain challenges. More specifically, the evolution of interactive media has made selling to consumers truly complex (Crittenden et al., 2010; Medrano et al., 2016). With the advent of the mobile channel, tablets, social media, and the integration of these new channels and devices in online and offline retailing, the landscape has continued to evolve, leading to profound changes in customer behavior (Verhoef et al., 2015).

A growing number of customers use multiple channels during their shopping journey. These kinds of shoppers are known as *omnishoppers*, and they expect a seamless experience across channels (Yurova et al., in press). For example, an omnishopper might research the characteristics of a product using a mobile app, compare prices on several websites from their laptop, and, finally, buy the product at a physical store. This consumer 3.0 uses new technology to search for information, offer opinions, explain experiences, make purchases, and talk to the brand. In an omnichannel environment, channels are used seamlessly and interchangeably during the search and purchase process, and it is difficult if not virtually impossible for retailers to control this use (Neslin et al., 2014; Verhoef et al., 2015).

Lu et al. (2005) consider mobile commerce to be the second wave of e-commerce. We believe that omnichannel commerce could be the third wave. Most studies on end-user beliefs and attitudes are conducted long after the systems have been adopted; while initial adoption is the first step in long-term usage, the factors affecting usage may not be the same as those influencing the initial adoption, or the degree of their effect may vary (Lu et al., 2005). Few papers have addressed the issue of pre-adoption criteria for omnishoppers, and explanations of why users behave in a particular way toward information technologies have predominantly focused on instrumental beliefs, such as perceived usefulness and perceived ease of use, as the drivers of usage intention. Previous papers in behavioral science and psychology suggest that holistic experiences (Schmitt, 1999) with technology, as captured in constructs such as enjoyment, flow, and social image, are potentially important explanatory variables in technology acceptance.

This paper aims to advance the theoretical understanding of the antecedents of omnishoppers' technology acceptance and use in relation to early adoption of omnichannel stores. To this end, it focuses on the acceptance and use of the technology that customers use in the “information prior to purchase” and “purchase” stages. We carried out this research in the fashion word, because it is one of the earliest industries to adopt this new strategy (PwC et al., 2016). This paper presents a new model of technology acceptance and use based on UTAUT2 (Venkatesh et al., 2012), extended to include two new dimensions—*personal innovativeness* and *perceived security*—and adapted to a specific context, i.e., the omnichannel environment.

Our research has important theoretical and managerial implications since studying the drivers of omnishoppers' shopping behavior would allow firms to follow different strategies in omnichannel customer management aimed at increasing customer satisfaction by offering an integrated shopping experience (Lazaris and Vrechopoulos, 2014; Neslin et al., 2014; Lazaris et al., 2015; Verhoef et al., 2015).

To achieve this goal, this paper proceeds as follows: first, we review the literature on the topic of omnichannel consumer behavior and the drivers of omnichannel shopping. Second, we develop a new theoretical model. Third, we describe and explain the empirical study. Fourth, we examine the results and implications of the findings and derive our conclusions. Fifth and finally, we address the limitations of the research and offer further research proposals.

## Literature review

### Omnichannel retailing context

Recent years have witnessed the emergence of new retailing channels. Thanks to new technologies, retailers can integrate all the information these channels provide, a phenomenon known as *omnichannel retailing* (Brynjolfsson et al., 2013).

The omnichannel concept is perceived as an evolution of multichannel retailing (Table 1). While multichannel retailing implies a division between the physical and online store, in the omnichannel environment, customers move freely among channels (online, mobile devices, and physical store), all within a single transaction process (Melero et al., 2016). *Omnis* is a Latin word meaning “all” or “universal,” so omnichannel means “all channels together” (Lazaris and Vrechopoulos, 2014). Because the channels are managed together, the perceived interaction is not with the channel, but rather the brand (Piotrowicz and Cuthbertson, 2014).

**Table 1:** **Multichannel vs. omnichannel**.

	**Multichannel strategy**	**Omnichannel strategy**
Concept	Division between the channels	Integration of all widespread channels
Degree of integration	Partial	Total
Channel scope	Retail channels: store, website, and mobile channel	Retail channels: store, website, mobile channel, social media, customer touchpoints
Customer relationship focus: brand vs. channel	Customer-retail channel focus	Customer-retail channel-brand focus
Objectives	Channel objectives (sales per channel, experience per channel)	All channels work together to offer a holistic customer experience
Channel management	Per channel	Cross-channel
	Management of channels and customer touchpoints geared toward optimizing the experience with each one	Synergetic management of the channels and customer touchpoints geared toward optimizing the holistic experience
	Perceived interaction with the channel	Perceived interaction with the brand
Customers	No possibility of triggering interaction	Can trigger full interaction
	Use channels in parallel	Use channels simultaneously
Retailers	No possibility of controlling integration of all channels	Control full integration of all channels
Sales people	Do not adapt selling behavior	Adapt selling behavior using different arguments depending on each customer's needs and knowledge of the product

*Source: Based on Rigby (2011), Piotrowicz and Cuthbertson (2014), Beck and Rygl (2015), and Verhoef et al. (2015)*.

The dominant characteristic of the omnichannel retailing phenomenon is that the strategy is centered on the customer and the customer's shopping experience, with a view to offering the shopper a holistic experience (Gupta et al., 2004; Shah et al., 2006).

In addition, the omnichannel environment places increasing emphasis on the interplay between channels and brands (Verhoef et al., 2015). Neslin et al. (2014) describe multiple purchase routes to show how this interplay works. Thus, not only is the omnichannel world broadening the scope of channels, it also integrates consideration of customer-brand-retail channel interactions.

Another important change is that the different channels are blurring together as the natural boundaries that once separated them begin to disappear. They are thus used seamlessly and interchangeably during the search, purchase, and post-purchase process, and it is difficult or virtually impossible for firms to control this usage (Verhoef et al., 2015).

### Consumer attitudes toward technology in an omnichannel context

Due to the increasing use of new technologies in retailing, consumer shopping habits and expectations are also changing. A new multi-device, multiscreen consumer has emerged who is better informed and demands omnichannel brands. Research has shown that omnichannel consumers are a growing global phenomenon (Schlager and Maas, 2013).

Customers expect a consistent, uniform, and integrated service or experience, regardless of the channel they use; they are willing to move seamlessly between channels—traditional store, online, and mobile—depending on their preferences, their current situation, the time of day, or the product category (Cook, 2014; Piotrowicz and Cuthbertson, 2014). The omnishopper no longer accesses the channel, but rather is always in it or in several at once, thanks to the possibilities offered by technology and mobility. These new shoppers want to use their own device to perform searches, compare products, ask for advice, or look for cheaper alternatives during their shopping journey in order to take advantage of the benefits offered by each channel (Yurova et al., in press). In addition, omnichannel consumers usually believe that they know more about a purchase than the salespeople and perceive themselves as having more control over the sales encounter (Rippé et al., 2015).

Despite the increase recorded in research on information and communication technology (ICT) and multichannel, it is important to continue investigating in the field of omnichannel consumer behavior (Neslin et al., 2014; Verhoef et al., 2015) and, especially, to determine how consumers' attitudes toward technology influence the purchasing decision process in the new context (Escobar-Rodríguez and Carvajal-Trujillo, 2014).

### Theory of acceptance and use of technology in an omnichannel context: model and hypothesis

Our research framework is based on an additional extension of the extended Unified Theory of Acceptance and Use of Technology (UTAUT2) model (Venkatesh et al., 2012) that seeks to identify the drivers of technology acceptance and use during the shopping journey to purchase in an omnichannel environment. Following the literature review, we chose the UTAUT2 model because it provides an explanation for ICT acceptance and use by consumers (Venkatesh et al., 2012). UTAUT2 is an extension of the original UTAUT model that synthesizes eight distinct theoretical models taken from sociological and psychological theories used in the literature on behavior (Table 2; Venkatesh et al., 2003). This theory contributes to the understanding of important phenomena such as, in this case, omnichannel consumers' attitudes toward technology and how they influence purchase intention in the shopping-process context. Under UTAUT2, a consumer's intention to accept and use ICT is affected by seven factors: *performance expectancy, effort expectancy, social influence, facilitating conditions, hedonic motivations, price value*, and *habit*.

**Table 2:** **Summary of models with constructs similar to those of UTAUT2**.

**Theory/model**	**Main constructs**	**Similar UTAUT2 construct**
Theory of reasoned action (TRA) (Fishbein and Ajzen, 1975)	Attitude toward behavior
	Subjective norm	SI
Technology acceptance model (TAM) (Davis, 1989; Davis et al., 1989)	Perceived usefulness	PE
	Perceived ease of use	EE
	Subjective norm	SI
Motivational model (MM) (Davis et al., 1992)	Extrinsic motivation	PE
	Intrinsic motivation
Theory of planned behavior (TPB) (Schifter and Ajzen, 1985; Ajzen, 1991)	Attitude toward behavior
	Subjective norm	SI
	Perceived behavioral control
Innovation diffusion theory (IDT) (Moore and Benbasat, 1991)	Relative advantage	PE
	Ease of use	EE
	Image	SI
	Visibility	FC
	Compatibility	
	Results demonstrability	
	Voluntariness of use	

*Source: Based on Escobar-Rodríguez and Carvajal-Trujillo, (2014). SI, Social Influence; PE, Performance Expectancy; EE, Effort Expectancy; FC, Facilitating conditions*.

As proposed by Venkatesh et al. (2012), UTAUT2 needs to be applied to different technologies and contexts, and other factors need be included, to verify its applicability, especially in the context of consumer behavior. To this end, building on previous work, in this study, we included *personal innovativeness* (San Martín and Herrero, 2012; Escobar-Rodríguez and Carvajal-Trujillo, 2014) and *perceived security* (Kim et al., 2008; Escobar-Rodríguez and Carvajal-Trujillo, 2014) to shed light on the degree to which the different factors included in the model influence consumers' purchase intentions.

#### The UTAUT2 model adapted to an omnichannel environment

As noted, our model was based on the UTAUT2 model.

*Performance expectancy* is defined as the degree to which using different channels and/or technologies during the shopping journey will provide consumers with benefits when they are buying fashion (Venkatesh et al., 2003, 2012). *Performance expectancy* has consistently been shown to be the strongest predictor of behavioral intention (e.g., Venkatesh et al., 2003, 2012; Escobar-Rodríguez and Carvajal-Trujillo, 2014) and purchase intention (Pascual-Miguel et al., 2015). In keeping with the literature, we proposed the following hypothesis:

1. H1. Performance expectancy positively affects omnichannel purchase intention.

*Effort expectancy* is the degree of ease associated with consumers' use of different touchpoints during the shopping process. Existing technology acceptance models include the concept of *effort expectancy* as *perceived ease of use* (TAM/TAM2) or *ease of use* (Innovation Diffusion Theory). According to previous studies (Karahanna and Straub, 1999), the *effort expectancy* construct is significant in both voluntary and mandatory usage contexts (Venkatesh et al., 2003) and positively affects purchase intention (Venkatesh et al., 2012). The following hypothesis was thus proposed for this construct:

1. H2. Effort expectancy positively affects omnichannel purchase intention.

*Social influence* is the extent to which consumers perceive that people who are important to them (family, friends, role models, etc.) believe they should use different channels depending on their needs. *Social influence*, understood as a direct determinant of behavioral intentions, is included as *subjective norm* in TRA, TAM2, and TPB, and as *image* in IDT (Fishbein and Ajzen, 1975; Schifter and Ajzen, 1985; Davis, 1989; Davis et al., 1989; Moore and Benbasat, 1991). The *social influence, subjective norm*, and *social norm* constructs all contain the explicit or implicit notion that individual behavior is influenced by how people believe others will view them as a result of having used the technology (Venkatesh et al., 2003) and positively affect purchase intention (Venkatesh et al., 2012). Therefore, the following hypothesis was proposed:

1. H3. Social influence positively affects omnichannel purchase intention.

*Habit* is defined as the extent to which people tend to perform behaviors automatically because of learning (Venkatesh et al., 2012). This concept, which was included as a new construct in the UTAUT2 model, has been considered a predictor of technology use in many studies (e.g., Kim et al., 2005; Kim and Malhotra, 2005; Limayem et al., 2007) and directly influences purchase intention (Venkatesh et al., 2012; Escobar-Rodríguez and Carvajal-Trujillo, 2014). Based on the literature, the following hypothesis was thus proposed:

1. H4. Habit positively affects omnichannel purchase intention.

In order to analyze consumers' motivations for adopting omnichannel behavior, we based our framework on the extended literature used in retailing. Previous research on shopping behavior suggests that customers use different channels at each stage of the shopping process to meet utilitarian and hedonic needs at the lowest cost relative to benefits, in other words, to maximize value (e.g., Balasubramanian et al., 2005; Noble et al., 2005; Konuş et al., 2008).

Shopping value can be both hedonic and utilitarian (Babin et al., 1994). Hedonic motivations are associated with adjectives such as *fun, pleasurable*, and *enjoyable* (e.g., Holbrook and Hirschman, 1982; Kim and Forsythe, 2007; To et al., 2007; Venkatesh et al., 2012). In contrast, utilitarian motivations are rational and task-oriented (Batra and Ahtola, 1991). Both dimensions are important because they are present in all shopping experiences and consumer behavior (Jones et al., 2006). Items such as clothing are classified in the highly hedonic product category due to their symbolic, experimental, and pleasing properties (Crowley et al., 1992). Consumers are more likely to select a physical store when they shop for hedonic fashion goods because strong physical environments elevate mood by providing opportunities for social interaction, product evaluation, and sensory stimulation (Nicholson et al., 2002). However, recent data show that consumers consider online fashion shopping to be a pleasurable activity and spend their leisure time searching for clothes using this medium (Blázquez, 2014).

In relation to technology acceptance and use, while *utilitarian motivation* was included as part of the *performance expectancy* construct in keeping with Venkatesh et al. (2003), *hedonic motivation* was included as a separate construct in UTAUT2 (Venkatesh et al., 2012). *Hedonic motivation* is defined as the fun or pleasure derived from using a technology, and it has been shown to play an important role in determining technology acceptance and use (Brown and Venkatesh, 2005). Numerous papers on ICT have demonstrated the influence of *hedonic motivation* on the intention both to use a technology and to purchase it (Van Der Heijden, 2004; Thong et al., 2006). Therefore, the following hypothesis was proposed:

1. H5. Hedonic motivations positively affect omnichannel purchase intention.

#### External variables applied in the extension of UTAUT2

When shoppers come into contact with a new technology or innovation, they have the opportunity to adopt or refuse it. Prior research has shown that innovative multichannel customers prefer to explore and use new alternatives (e.g., Steenkamp and Baumgartner, 1992; Rogers, 1995; Konuş et al., 2008). In addition, several studies in the e-commerce literature have demonstrated the important role that innovativeness plays in purchase intention in different contexts (e.g., Herrero and Rodriguez del Bosque, 2008; Lu et al., 2011; San Martín and Herrero, 2012; Escobar-Rodríguez and Carvajal-Trujillo, 2014).

*Personal innovativeness* is defined as the degree to which a person prefers to try new and different products or channels and to seek out new experiences requiring a more extensive search (Midgley and Dowling, 1978). Many papers have highlighted that consumer innovativeness is a highly influential factor in ICT adoption and on purchase intention (e.g., Agarwal and Prasad, 1998; Citrin et al., 2000; Herrero and Rodriguez del Bosque, 2008; San Martín and Herrero, 2012; Escobar-Rodríguez and Carvajal-Trujillo, 2014). The following research hypothesis was thus formulated:

1. H6. Personal innovativeness positively affects omnichannel purchase intention.

Additionally, we included the *perceived security* of the online channels, referring to the belief that the Internet is a secure option for sending personal data (Escobar-Rodríguez and Carvajal-Trujillo, 2014; Bonsón Ponte et al., 2015). *Perceived security* can be defined as the perception by consumers that the omnichannel companies' technology strategies include the antecedents of information security, such as authentication, protection, verification, or encryption (Kim et al., 2008). If consumers perceive that the online channels have security attributes, they will deduce that the retailer's intention is to guarantee security during the purchasing process (Chellappa and Pavlou, 2002). There is some evidence that the *perceived security* of online channels positively affects the intent to purchase using these kind of channels (e.g., Salisbury et al., 2001; Frasquet et al., 2015). In light of these findings, it was hypothesized that *perceived security* is related to purchase intention as follows:

1. H7. Perceived security positively affects the omnichannel purchase intention.

**Figure 1:**
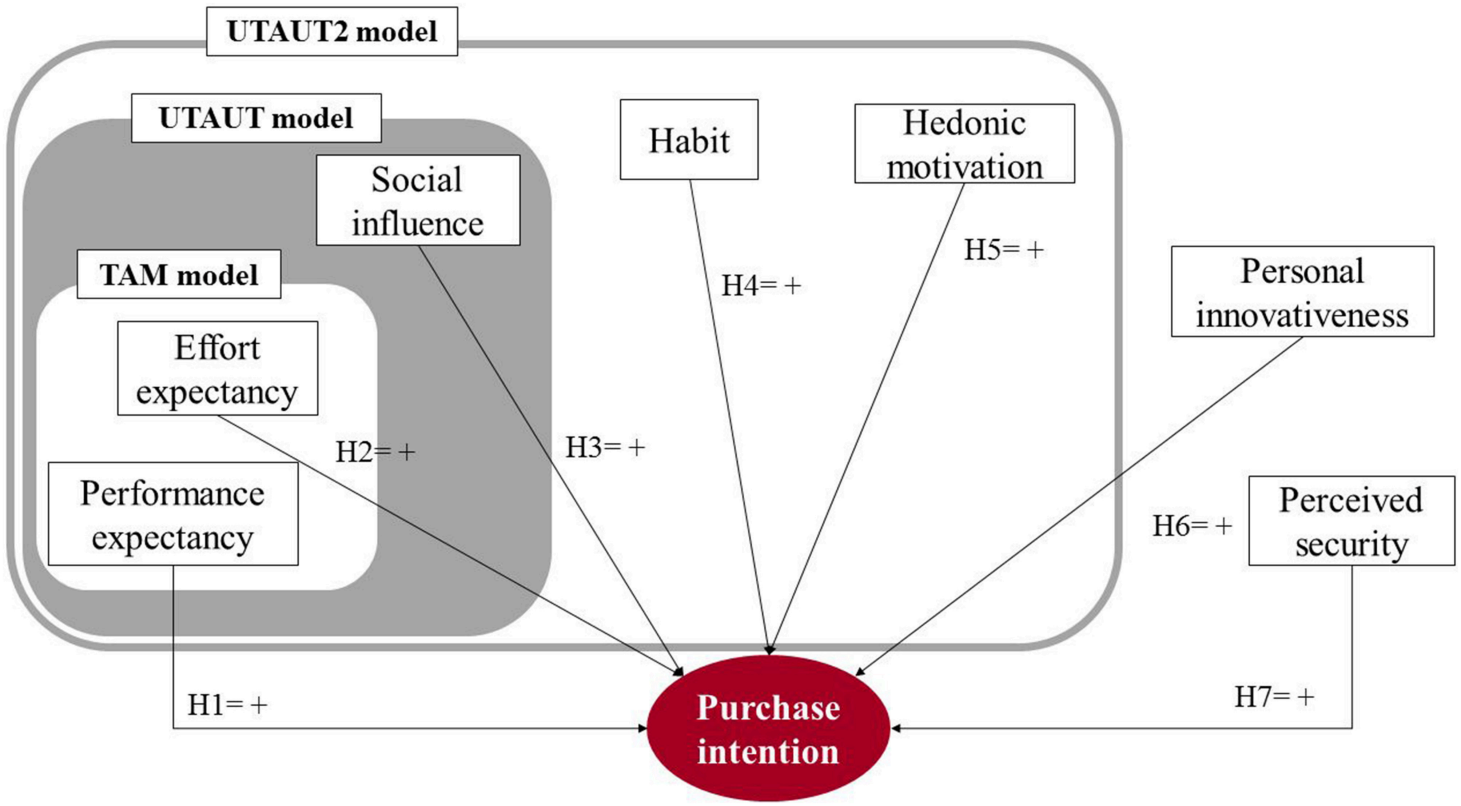
**Theoretical model of purchase intention in an omnichannel store**.

Figure 1 shows the theoretical model based on the seven hypotheses, reflecting how the antecedents of technology acceptance and use affect purchase intention in an omnichannel environment.

## Methodology

We designed an online survey focused on omnichannel fashion retail customers. The questionnaire was administered to a Spanish Internet panel. For the purposes of our study, we defined *omnichannel shoppers* as those shoppers who use at least two channels of the same retailer during their shopping journey. The panelists were screened to select those members that fit our definition of omnichannel shoppers. In all, 628 respondents indicated their behavior with regard to their most recent purchase in the 12 months prior to the collection of the data (January 2016).

To carry out the study we selected the company Zara for several reasons. First, Zara is one of the most well-known and important fashion retailers. Additionally, the brand follows an omnichannel strategy, allowing its customers to combine different online channels (the company website, social media, and the mobile app) with the offline channels throughout their customer journey. In other words, shoppers can search for information on a product using the Zara mobile app, buy the product on the Zara website (www.zara.com), and then pick up or return the product at the physical store. However, the most important reason for choosing a single company to study the factors influencing omnichannel customers' behavior was to isolate the omnichannel factor, that is, we wanted to determine the drivers for using different channels and/or technologies of a single company during a single shopping process.

The questionnaire consisted of two parts. The first part contained statements about shopping motives. Based on their most recent shopping process (Table 3), respondents were instructed to rate their agreement with each item on a seven-point Likert scale ranging from 1 (strongly agree) to 7 (strongly disagree).

**Table 3:** **Theory of use and acceptance of technology in an omnichannel context**.

**Dimension**	**Item and definition**
Hedonic motivations (Childers et al., 2001)	Hedonic1. Being able to use multiple channels throughout the customer journey is enjoyable
	Hedonic2. Being able to use multiple channels throughout the customer journey is pleasurable
	Hedonic3. Being able to use multiple channels throughout the customer journey is interesting
Performance expectancy (Venkatesh et al., 2003)	Performance1. Being able to use multiple channels throughout the customer journey allows me to purchase quickly
	Performance2. Being able to use multiple channels throughout the customer journey is useful to me
	Performance3. Being able to use multiple channels throughout the customer journey makes my life easier
Effort expectancy (Venkatesh et al., 2003)	Effort1. I find Zara's different online platforms (website and mobile app) easy to use
	Effort2. Learning how to use Zara's different online platforms (website and mobile app) is easy for me
Social influence (Venkatesh et al., 2003)	Social1. People who are important to me think that I should use different channels, choosing whichever is most convenient at any given time
	Social2. People who influence my behavior think that I should use different channels, choosing whichever is most convenient at any given time
	Social3. People whose opinions *I*-value prefer that I use different channels, choosing whichever is most convenient at any given time
	Social4. People whose opinions *I*-value use different channels, choosing whichever is most convenient at any given time
Habit (Limayem and Hirt, 2003; Venkatesh et al., 2012)	Habit1. The use of different channels (physical store, website, mobile app) throughout the customer journey has become a habit for me
	Habit2. I frequently use different channels throughout the customer journey
Security (Cha, 2011)	Security1. Using credit cards to make purchases over the Internet is safe
	Security2. Making payments online is safe
	Security3. Giving my personal data to Zara seems safe
Innovativeness (Goldsmith and Hofacker, 1991; Lu et al., 2005)	Innovativeness1.When I hear about a new technology, I search for a way to try it
	Innovativeness2. Among my friends or family, I am usually the first to try new technologies
	Innovativeness3. Before testing a new product or brand, I seek the opinion of people who have already tried it
	Innovativeness4. I like to experiment and try new technologies
Expected behavior	
Purchase intention (Pantano and Viassone, 2015)	PI1. I would purchase in this kind of store
	PI2. I would tell my friends to purchase in this kind of store
	PI3. I would like to repeat my experience in this kind of store

The second part of the questionnaire was used to gather sociodemographic information, such as gender, age, employment status, and education (Table 4). The sample was highly representative of the distribution of online shoppers according to recent surveys (Corpora 360 and iab Spain, 2015).

**Table 4:** **Technical details of the data collection and sample description**.

**Universe**	**People who used at least two channels during their shopping journey**	
Sample procedure	Stratified by gender and age
Data collection	Online survey	
Study area	Spain	
Sample size	628 people	
Date of fieldwork	January 2016	
Sample characteristics		
		**Sample %**
Gender	Male	49.2
	Female	50.8
Age	16–24	13.4
	25–34	37.7
	35–44	32.0
	45–54	12.9
	55+	4.0
Occupation	Student	9.4
	Homemaker	4.1
	Unemployed	10.2
	Retired	1.4
	Self-employed	12.7
	Employee	62.1
Education	Low level of education	3.5
	High school	47.6
	College	48.9
Omnichannel shopper	Used 2 channels	81.0
	Used 3 channels	11.8
	Used 4 channels	7.2

Because of the novelty of the field of application, the measurement scales were then translated into Spanish using a back-translation method, whereby one person translated the items into Spanish and two others translated them back into English, making it possible to check for any misunderstandings or misspellings resulting from the translation (Brislin, 1970). In addition, we conducted a pretest with 25 participants to ensure the comprehensibility of the questions.

We used IBM SPSS Statistics 19 to perform the exploratory factor analysis. Subsequently we undertook a regression analysis of latent variables based on the partial least squares (PLS) technique.

The aim of this research was to explore technology acceptance and use in an omnichannel context. To achieve this aim, fundamentally, theory development, we chose to use the PLS technique to evaluate the structural model before testing the causal model. Next, we estimated a confirmatory factor model to study the validity of the scale and examined the underlying structure. To this end, we created a causal model and used structural equations to evaluate the scale and the effect of technology acceptance and use on omnichannel shoppers' purchase intentions.

The study was approved by the Head of Ethics at the Faculty of Business Administration of La Rioja University. All participants provided informed consent.

## Data analysis and results

### Measurement model

We performed a confirmatory factor analysis to which we made a few amendments. It was likewise verified that the loadings of all the standardized parameters were greater that 0.7 (Hair et al., 2013). The item *innovativeness3* had a value lower that 0.7 and a *t*-value lower that 1.96. We thus decided to exclude it to improve the model's convergence, as recommended by Anderson and Gerbing (1988). The model confirms that the indicators converge with the assigned factors.

The model was verified in terms of construct reliability (i.e., composite reliability and Cronbach's alpha), convergent validity, and discriminant validity. The composite reliability and Cronbach's alpha values were >0.70, and the constructs' convergent validity was also confirmed, with an average variance explained (AVE) >0.50 in all cases. The discriminant validity of the constructs was measured by comparing the square root of the AVE of each construct with the correlations between constructs (Roldán and Sánchez-Franco, 2012). The square root of the AVE (diagonal elements in italics in Table 5) had to be larger than the corresponding inter-construct correlation (off-diagonal elements in Table 5). This criterion was also met in all cases. Furthermore, each item's loading on its corresponding factor was greater than the cross-loadings on the other factors.

**Table 5:** **Construct reliability, convergent validity, and discriminant validity**.

	**CR > 0.7**	**α**	**AVE > 0.5**	**EE**	**H**	**HM**	**PI**	**PE**	**PS**	**SI**	**PUR_IN**
EE	0.93	0.86	0.88	*0.94*							
H	0.93	0.86	0.87	0.45	*0.93*						
HM	0.93	0.89	0.82	0.58	0.58	*0.90*					
PI	0.92	0.86	0.78	0.49	0.51	0.54	*0.89*				
PE	0.92	0.87	0.80	0.62	0.53	0.70	0.51	*0.89*			
PS	0.93	0.89	0.82	0.54	0.51	0.44	0.43	0.46	*0.90*		
SI	0.96	0.94	0.85	0.45	0.67	0.60	0.53	0.52	0.55	*0.92*	
PUR_IN	0.95	0.92	0.86	0.57	0.40	0.51	0.57	0.58	0.41	0.43	*0.93*

*EE, Effort Expectancy; H, Habit; HM, Hedonic Motivation; PI, Personal Innovativeness; PE, Performance Expectancy; PS, Perceived Security; SI, Social Influence; PUR_IN, Purchase Intention*.

### Structural model

Bootstrapping with 5000 resamples was used to assess the significance of the path coefficients obtained by PLS-SEM (Hair et al., 2011). The model explains the intention to purchase in the omnichannel context well, with an *R*^2^ of 47.9% (Table 6). Stone-Geisser's cross-validated redundancy *Q*^2^ was >0, specifically, 0.406. This result confirmed the predictive power of the proposed model (see Hair et al., 2011).

**Table 6:** **Results of the structural model**.

	***R*^2^**	***Q*^2^**	**Path coeff**.	***t***	**Low CI**	**High CI**	**Explained variance%**	***P*-values**	**Hypotheses**
	47.9%	0.406							
PE –> PUR_IN			0.238	4.191	0.123	0.342	13.80	0.000	H1: Accepted
EE –> PUR_IN			0.255	4.953	0.157	0.356	14.54	0.000	H2: Accepted
SI –> PUR_IN			0.025	0.490	−0.077	0.125	1.08	0.624	H3: Rejected
H –> PUR_IN			−0.048	0.937	−0.145	0.059	−1.92	0.349	H4: Rejected
HM –> PUR_IN			0.034	0.572	−0.080	0.155	1.73	0.567	H5: Rejected
PI –> PUR_IN			0.310	6.506	0.224	0.409	17.67	0.000	H6: Accepted
PS –> PUR_SE			0.023	0.467	−0.078	0.122	0.94	0.640	H7: Rejected

*PE, Performance Expectancy; EE, Effort Expectancy; SI, Social Influence; H, Habit; HM, Hedonic Motivation; PI, Personal Innovativeness; PS, Perceived Security; PUR_IN, Purchase Intention*.

The sign, magnitude, and significance of the path coefficients are shown in Table 6. Three hypotheses were supported by the results: H1 (regarding the influence of *performance expectancy*), H2 (regarding the influence of *effort expectancy*), and H6 (regarding the influence of *personal innovativeness*). In contrast, H3 (regarding social influence), H4 (regarding the influence of *habit*), H5 (regarding the influence of *hedonic motivation*), and H7 (regarding the influence of *perceived security*) were rejected, as the relationships were not significant.

## Conclusion and managerial implications

Today's increasingly competitive retail world has given rise to a new phenomenon known as *omnichannel retailing* (e.g., Rigby et al., 2012; Neslin et al., 2014; Beck and Rygl, 2015; Verhoef et al., 2015). This phenomenon can be defined as the customer management strategy throughout the life cycle of the customer relationship whereby the shopper interacts with the brand through different devices and channels (mainly the physical store, the online channel, the mobile channel, and social media), and, thus, all touchpoints must be integrated to provide a seamless and complete shopping experience, regardless of the channel used. Omnichannel retailing stands to become the third wave of e-commerce.

Few studies have analyzed the antecedents of omnishopper behavior (e.g., Lazaris et al., 2014; Neslin et al., 2014; Verhoef et al., 2015). The main goal of the present research was to identify the drivers of technology acceptance and use among omnichannel consumers and to analyze how they affect purchase intention in an omnichannel context. To this end, we proposed a new model based on the extended Unified Theory of Acceptance and Use of Technology (UTAUT2) model (Venkatesh et al., 2012), which we further extended to include two new factors: *personal innovativeness* and *perceived security*. Both *personal innovativeness* and *perceived security* have been found to be important for the adoption of new technologies in the literature on consumer behavior (e.g., Salisbury et al., 2001; Herrero and Rodriguez del Bosque, 2008; Escobar-Rodríguez and Carvajal-Trujillo, 2014; Frasquet et al., 2015). The present paper helps to advance the theoretical understanding of the antecedents of consumer 3.0 technology acceptance and use in the early adoption of omnichannel stores.

The model was found to predict omnichannel purchase intention (*R*^2^ = 47.9%). Our findings show that a consumer's intention to purchase in an omnichannel store is influenced by *personal innovativeness, effort expectancy*, and *performance expectancy*. In contrast, contrary to our hypotheses based on the broader previous literature, *habit, hedonic motivation, social influence*, and *perceived security* do not affect omnichannel purchase intention.

*Personal innovativeness* is the strongest predictor of purchase intention in the omnichannel context. This factor plays an important role as a direct driver of omnichannel purchase intention. This finding is consistent with those of previous papers (e.g., Herrero and Rodriguez del Bosque, 2008; Lu et al., 2011; San Martín and Herrero, 2012; Escobar-Rodríguez and Carvajal-Trujillo, 2014). Thus, individuals who are more innovative with regard to ICT will have a stronger intention to purchase using different channels and devices in an omnichannel environment. Our findings show that omnishoppers seek out new technology in order to experiment with it and be the first to try it among their family and friends. Managers should thus take this technological profile into account and constantly roll out new technologies in different ways in order to attract and surprise these kinds of shoppers.

Our findings also show that *effort expectancy* and *performance expectancy* are significant factors in explaining attitude and purchase intention, with a positive effect on behavioral intention, as has been widely reported in the literature (e.g., Childers et al., 2001; Verhoef et al., 2007; Rose et al., 2012). *Effort expectancy* is the second strongest predictor and has a direct positive influence on purchase intention (e.g., Karahanna and Straub, 1999; Venkatesh et al., 2003, 2012). This could be because omnishoppers are more used to using multiple channels and are more task-oriented, using different channels or technologies to look for better prices or maximize convenience at any given time. In keeping with previous research (e.g., Venkatesh et al., 2003, 2012; Escobar-Rodríguez and Carvajal-Trujillo, 2014), *performance expectancy* was found to be the third strongest predictor of behavioral intention in an omnichannel environment.

Although the literature has recognized the influence of normative factors such as *social influence* on people's attitude, intentions, and behavior (e.g., Fishbein and Ajzen, 1975; Bagozzi, 2000; Venkatesh et al., 2012), our results show that this factor does not influence the intention to purchase in an omnichannel environment. On the contrary, in line with previous work (e.g., Casaló et al., 2010; San Martín and Herrero, 2012), *social influence* was found not to affect purchase intention. This finding contrasts with those reported elsewhere (Kim et al., 2009; Venkatesh et al., 2012; Escobar-Rodríguez and Carvajal-Trujillo, 2014; Pelegrín-Borondo et al., 2016). This may be because technology use is not conditioned by other people's opinions; it could also be due to the specific sector under study. In either case, it is a topic that should be studied further.

Contrary to previous studies (e.g., Venkatesh et al., 2012; Escobar-Rodríguez and Carvajal-Trujillo, 2014), our results indicate that *habit* does not influence omnichannel purchase intention. This could be because customers are not used to using different channels due to the relatively low number of companies that allow customers to use multiple channels simultaneously. In keeping with authors such as Valentini et al. (2011) and Melero et al. (2016), we believe this variable will increase in importance in the coming years, as more and more retailers implement true omnichannel strategies.

In our research, the hypothesized influence of *hedonic motivation* on purchase intention was found to be low. Previous work in other contexts has found a positive relationship between these variables (e.g.,Van Der Heijden, 2004; Thong et al., 2006; Venkatesh et al., 2012; Escobar-Rodríguez and Carvajal-Trujillo, 2014). These findings are probably because, when omnishoppers use different channels and touchpoints, they expect a seamless, holistic experience throughout their shopping journey. In other words, hedonic and utilitarian motivation are part of the same construct (Melero et al., 2016). In addition, technology acceptance and use is more of a new experience related to the innovativeness profile than a hedonic one, i.e., excitement over discovering how something will work rather than expected enjoyment based on prior experience.

Finally, contrary to previous findings (e.g., Salisbury et al., 2001; Frasquet et al., 2015), *perceived security* did not influence omnichannel purchase intention. We interpreted these results to mean that the possibility of buying in an omnichannel context offsets the influence of the need for security, an important factor in e-commerce, by offering the option of traditional in-store payment, which nullifies the effect of perceived risk in e-commerce. In this sense, omnichannel stores offer an opportunity to attract more conservative consumers who perceive an increased risk in e-commerce to a more interactive scenario in which retailers can use new technologies to manage customer relationships based on direct contact in the physical store.

Our study contributes to the current literature on omnichannel consumer behavior by adapting the previous UTAUT models to include two new factors in order to determine how the technologies used during the shopping process affect the intention to purchase in an omnichannel context. The results have practical implications for omnichannel retailer managers regarding the best management and marketing strategies for improving a key part of their business, namely, the creation of a holistic shopping experience for their customers (Lemon and Verhoef, in press). Specifically, retailers need to properly define not only which technologies they will invest in, but also how they will encourage the acceptance thereof, as this acceptance is an important predictor of purchase intention. In particular, in-store technology has to be focused on creating a new integrated customer experience, using technology that is practical, enjoyable, and interesting in order to ensure that innovative customers perceive that the new omnichannel stores facilitate and expedite their shopping journey.

Our paper has some limitations. Our data are related to consumer behavior in a particular case: the buying process in the fashion retailer Zara. It would be interesting to replicate this study in another product category or country to compare the results.

Our research also suggests interesting lines of future research, such as identifying omnichannel consumer profiles in order to personalize the customer shopping experience. Likewise, future studies could investigate the new role of technology in the physical store in an omnichannel environment. In addition, the influence of sociodemographic variables, such as age or gender, as moderator variables to complement the current model should be explored. In keeping with Chiu et al. (2012), we think it would also be interesting to examine *habit* as a moderator variable in purchase intention.

Finally, fashion companies need to determine which factors matter most to consumers 3.0 when they set out on their shopping journey in order to adapt their strategies to shoppers' motivations. This study has sought to shed light on the new omnichannel phenomenon. Technology is changing the future of retailing. The key will lie in successfully integrating all channels in order to think about them as consumers do and try to offer shoppers an integrated and comprehensive shopping experience.

## Author contributions

The three authors have equally participated in literature review, data analysis and writing of the paper.

### Conflict of interest statement

The authors declare that the research was conducted in the absence of any commercial or financial relationships that could be construed as a potential conflict of interest.
